# Development of an algorithm for determination of the likelihood of virological failure in HIV-positive adults receiving antiretroviral therapy in decentralized care

**DOI:** 10.1080/16549716.2017.1371961

**Published:** 2017-09-15

**Authors:** Anton Reepalu, Taye Tolera Balcha, Sten Skogmar, Per-Erik Isberg, Patrik Medstrand, Per Björkman

**Affiliations:** ^a^ Clinical Infection Medicine, Department of Translational Medicine, Lund University, Malmö, Sweden; ^b^ Armauer Hansen Research Institute, Addis Ababa, Ethiopia; ^c^ Department of Statistics, Lund University, Lund, Sweden; ^d^ Clinical Virology, Department of Translational Medicine, Lund University, Malmö, Sweden

**Keywords:** Human immunodeficiency virus, viral load, criteria, Ethiopia, CD4 lymphocyte count

## Abstract

**Background**: Early identification of virological failure (VF) limits occurrence and spread of drug-resistant viruses in patients receiving antiretroviral treatment (ART). Viral load (VL) monitoring is therefore recommended, but capacities to comply with this are insufficient in many low-income countries. Clinical algorithms might identify persons at higher likelihood of VF to allocate VL resources.

**Objectives**: We aimed to construct a VF algorithm (the Viral Load Testing Criteria; VLTC) and compare its performance to the 2013 WHO treatment failure criteria.

**Methods**: Subjects with VL results available 1 year after ART start (n = 494) were identified from a cohort of ART-naïve adults (n = 812), prospectively recruited and followed 2011–2015 at Ethiopian health centres. VF was defined as VL≥1000 copies/mL. Variables recorded at the time of sampling, with potential association with VF, were used to construct the algorithm based on multivariate logistic regression.

**Results**: Fifty-seven individuals (12%) had VF, which was independently associated with CD4 count <350 cells/mm^3^, previous ART interruption, and short mid-upper arm circumference (<24cm and <23cm, for men and women, respectively). These variables were included in the VLTC. In derivation, the VLTC identified 52/57 with VF; sensitivity 91%, specificity 43%, positive predictive value (PPV) 17%, negative predictive value (NPV) 97%. In comparison, the WHO criteria identified 38/57 with VF (sensitivity 67%, specificity 74%, PPV 25%, NPV 94%).

**Conclusions**: The VLTC identified subjects at greater likelihood of VF, with higher sensitivity and NPV than the WHO criteria. If external validation confirms this performance, these criteria could be used to allocate limited VL resources. Due to its limited specificity, it cannot be used to determine treatment failure in the absence of a confirmatory viral load.

## Background

Regular HIV-RNA quantification in plasma (viral load; VL) is the most accurate method to monitor antiretroviral treatment (ART), and has been routinely used in HIV care in high-income countries since ART became available [1,2]. VL monitoring allows for early detection of virological failure (VF) before clinical disease progression and accumulation of resistance mutations has occurred [3–5]. Viral load results can also be used for adherence counselling, and may save costs by preventing unnecessary switches to 2nd line ART [6,7]. For these reasons, the WHO recommends regular VL monitoring, at 6 and 12 months after ART start and annually thereafter, for all people receiving ART [8].

In 2015, 46% of the 36.9 million people living with HIV (PLHIV) in the world, of whom the majority reside in Sub-Saharan Africa, had started ART [9]. This achievement has been made possible by decentralisation and integration of HIV care into primary health care. In these settings, access to VL monitoring is severely restricted [10], and expansion of viral load capacities is hampered by high cost and technical requirements [11].

In several fields of medicine algorithms are used to determine the likelihood of certain conditions being present, to target further investigations. This approach is also used in HIV care, especially for estimation of the risk of tuberculosis co-infection [12,13]. Some groups have also attempted to develop algorithms for determination of VF [14–17], but to our knowledge these algorithms are hitherto not in general use nor recommended in ART guidelines.

In areas where viral load monitoring is not available the WHO recommends using clinical and/or immunological criteria to identify patients failing on treatment [8]. However, these criteria are not evidence-based, and have poor performance [18]. Alternative strategies for detection of treatment failure are therefore required for ART programs in low-income countries until universal VL monitoring is established.

In Ethiopia, nearly 400,000 out of an estimated 781,000 PLHIV had started ART by 2016 [19,20], with most HIV care provided through health centres. Until 2015, viral load testing was only recommended for cases of clinically suspected treatment failure [21]. Although annual viral load testing is currently recommended for all patients receiving ART, the resources to comply with this are limited. The use of algorithms to prioritize patients for viral load testing should therefore be considered to optimize use of available laboratory resources.

For this purpose, we have constructed an algorithm intended for use in decentralized HIV care settings to identify subjects with increased likelihood of VF who need further evaluation with VL testing. The algorithm is based on robust variables independently associated with VF in cohort of adults receiving care at Ethiopian health centres. The performance of the algorithm is compared with the 2013 WHO failure criteria in our cohort participants.

## Methods

### Patient population

This study is based on a patient cohort prospectively recruited from October 2011 until March 2013 at all five public health centres providing ART in and around the city of Adama, Ethiopia (uptake area 600 000 inhabitants). The cohort was recruited to study methods to diagnose tuberculosis and virological failure in HIV positive adults. Detailed descriptions of the cohort has been published previously [22,23].

ART-naïve patients aged ≥18 years with recorded CD4 cell count <350 cells/mm^3^ and/or WHO stage IV disease were eligible for enrolment in the cohort. Subjects with previous ART experience and/or tuberculosis treatment for >2 weeks were excluded.

At inclusion, socio-demographic and medical information was collected, and at all subsequent visits symptoms and clinical findings were recorded following structured questionnaires. All patients enrolled in HIV care in Ethiopia receive adherence counselling at least twice before starting ART and adherence assessments are made at all clinical visits after ART initiation [21]. For study purposes, medication adherence was estimated using a three-question panel on: punctuality of daily tablet intake, number of missed doses weekly, and duration since last missed dose [24]. Treatment interruption of ART since last visit (for any reason and at least one day’s duration) was also recorded.

Follow-up visits after ART start were scheduled at months 1, 2, 3, 6, 9, 12, and biannually thereafter. Blood sampling for haematological parameters, CD4 cell counts and storage of plasma for later VL testing was performed in all participants at months 1, 3, 6, 12, and subsequent visits. Participants could, however, decline to give blood without being excluded from the study.

HIV-1 RNA quantification was performed on plasma aliquots stored at −80°C at the regional laboratory in Adama using Abbott Real-Time HIV-1 assay (Abbott Molecular Inc., Des Plaines, IL; detection limit 40 copies/mL) in batches during the study period. Results were communicated to care providers with recommendations to assess adherence and repeat viral load testing on subjects with VL ≥1000 copies/mL before referral for second line ART (according to national guidelines). Blood sampling was performed at the same visit as recording of symptoms and clinical findings, thereby blinding the clinicians performing the examinations with regard to VF. External quality assurance of the regional laboratory is regularly performed by the Center for Disease Control and Prevention (Atlanta, GA).

Subjects with a study visit 1 year (9–15 months) after ART start, with an accompanying viral load result, were included in this study.

### The WHO criteria

The WHO 2013 clinical failure criterion is defined as a new or recurrent clinical event indicating severe immunodeficiency after 6 months of ART, whereas immunological failure is defined as a CD4 count below or at the value measured before starting ART or a CD4 count <100 cells/mm^3^ [25]. In this study, all stage 3 and 4 events were considered to indicate severe immunodeficiency.

### Statistical analysis

The aim of this study was to construct an algorithm with high sensitivity and acceptable specificity to identify subjects with VF 1 year after ART start. We used VL ≥1000 copies/mL as definition of VF.

To construct the algorithm, all variables registered at the 12-month visit were assessed for possible association with VF. Variables had to be considered robust with potential to be used in a decentralized care setting to be included. Since active case-finding for tuberculosis had been performed on the cohort at inclusion we included this parameter to evaluate its potential impact on VF.

We used routinely used threshold levels to dichotomize body-mass index (BMI; <18.5 kg/m^2^), mid-upper arm circumference (MUAC; <23 cm for women and <24 cm for men), haemoglobin (<11.0 g/dL), and lymphocyte count (<1100 cells/mm^3^) [26,27]. Karnofsky performance status (KPS), and CD4 count were analysed with ROC (receiver operating characteristics) curves and dichotomised at maximum sensitivity with acceptable specificity; KPS at <90% and CD4 count at <350 cells/mm^3^. Age was used as a continuous variable since no clinically useful threshold could be determined.

All variables were analysed with univariate logistic regression. Variables associated with VF in univariate analysis (p < 0.3) were entered into a multivariate regression model followed by stepwise backward removal of the least significant variable at each step until only variables independently associated with VF (p < 0.05) remained. The remaining variables constituted the VLTC.

The diagnostic accuracy of the VLTC and WHO criteria were evaluated using sensitivity, specificity, positive predictive value (PPV), and negative predictive value (NPV) with 95% CI. To further describe the discriminatory potential, numbers needed to test to identify one subject with VF (NNT) was calculated.

To assess possible effect modification (interaction) between the algorithm and gender, the performance of the algorithm and its individual components were also analysed separately for men and women.

We performed a sensitivity analysis including only subjects with complete data for all criteria included in the WHO criteria and VLTC, and assessed possible effects on the performance of the algorithms due to missing data. In this study, all participants underwent active case-finding for active tuberculosis at inclusion. To assess the impact of these investigations on the WHO clinical criteria, which includes incident tuberculosis during ART, subjects with tuberculosis were excluded in an additional sensitivity analysis. The statistical analyses were performed in SPSS version 21 (IBM Corp, Armonk, NY).

## Results

### Patient characteristics

**Figure 1.**
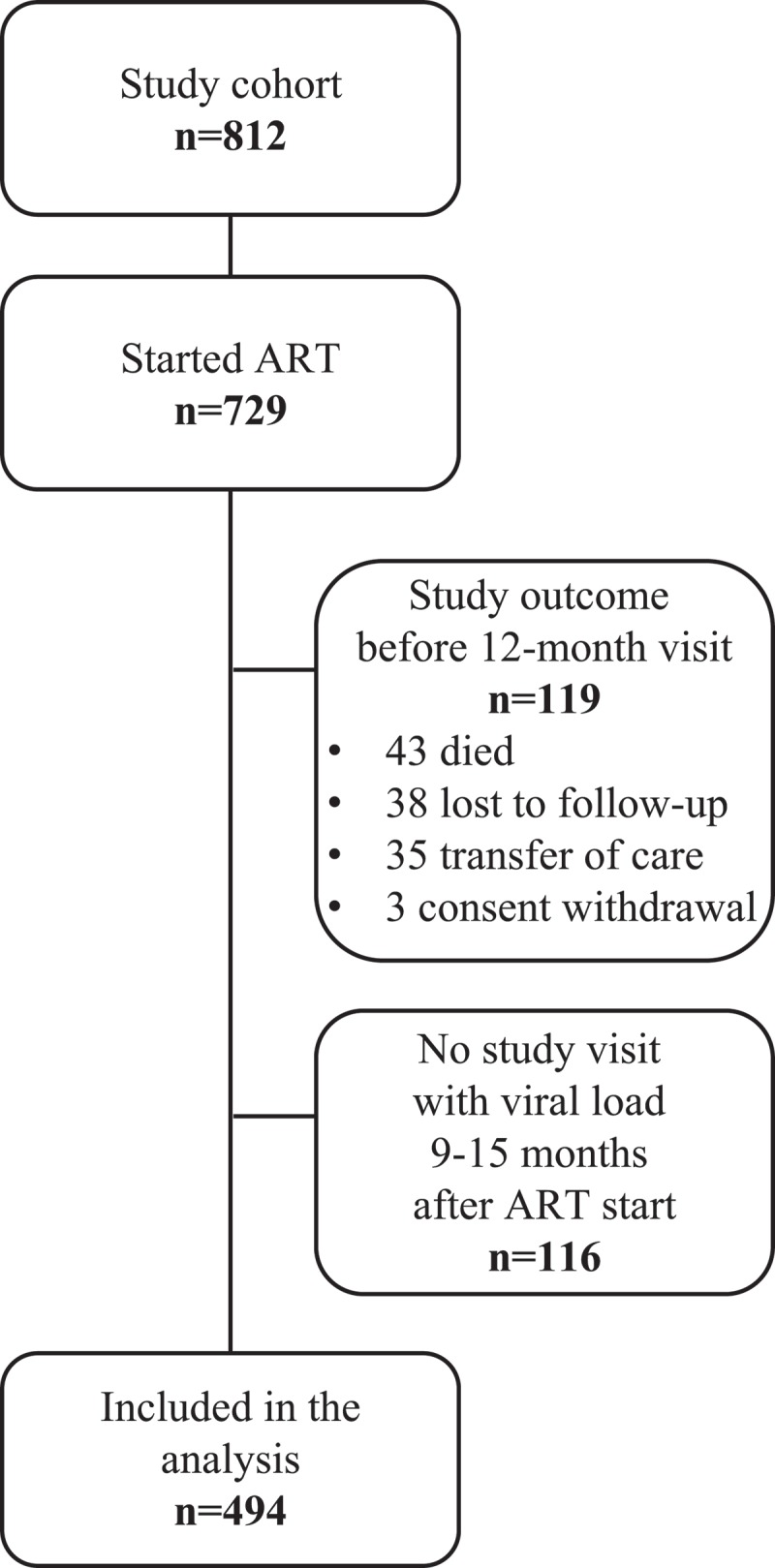
Flow chart of study participants.

Among the 812 individuals in the cohort, 729 (90%) started ART during the follow-up period, Figure 1. Baseline characteristics are shown in Table 1. Patients not remaining in the study until the 12-month visit could not be included in this study. A similar number of men and women did not remain in follow-up due to death (22 men and 21 women) or loss to follow-up (20 men and 19 women). Participants without viral load results and/or no registered study visit within the defined time frame 1 year after ART start, 116/610 (19%), were excluded. Characteristics of excluded participants were similar to those of included participants, except for a greater proportion of men being excluded due to unavailable data, Table 1. Seventy-five of the 82 participants without 12-month data remained in follow-up at 18 months of ART (2 were transferred out, 3 were lost to follow-up, and 2 declined further participation).

**Table 1.** Baseline characteristics of study participants.

Characteristic	Started ART (n = 729)	12-month data available (n = 494)	12-month data unavailable (n = 116)	*P*
Age, years	32 (28–40)	33 (28–40)	30 (28–38)	0.13
Female	431 (59)	313 (63)	56 (48)	<0.01
BMI, kg/m^2^	19 (18–21)	20 (18–22)	19 (18–21)	0.51
MUAC, cm	23 (21–25)	23 (21–25)	23 (21–25)	0.59
Karnofsky status, %	80 (80–90)	90 (80–90)	90 (80–90)	0.37
WHO clinical stage				0.68
Stage I	131 (18)	100 (20)	18 (16)	
Stage II	207 (28)	144 (29)	34 (29)	
Stage III	313 (43)	201 (41)	52 (45)	
Stage IV	77 (11)	48 (10)	12 (10)	
CD4 count, cells/mm^3^	187 (116–274)	192 (127–274)	199 (115–302)	0.64
Lymphocyte count, cells/mm^3^	1400 (1100–1800)	1400 (1100–1900)	1400 (1000–1800)	0.93
Haemoglobin, g/dL	11.5 (10.2–12.7)	11.6 (10.3–12.7)	11.6 (10.3–13.0)	0.93
Active tuberculosis at baseline	137 (19)	88 (18)	23 (21)	0.61

Data presented as n (%) of patients or median value (interquartile range).

*P* calculated with Chi-square and Mann-Whitney U test for categorical and continuous variables, respectively.

Abbreviations: ART, antiretroviral therapy; BMI, body-mass index; MUAC, mid-upper arm circumference.

In total, 57 of the included 494 participants (12%) met our definition of VF 1 year after starting ART.

### Derivation of the VLTC

In univariate analysis, the following variables were associated with VF (p < 0.3): gender, age, KPS, BMI, gender-specific MUAC, previous ART interruption, CD4 count <350 cells/mm^3^ haemoglobin and lymphocyte count, supplemental Table 1. Tuberculosis at inclusion, adherence <95%, and the clinical sign skin rash, did not show any association with VF.

After stepwise removal from the multivariate model the following variables remained: gender-specific MUAC, CD4 count <350 cells/mm^3^, and previous ART interruption, Table 2. Age was kept in the multivariate model for adjustments due to its univariate association with VF but without any clear threshold level.

**Table 2.** Variables associated with virological failure in multivariate analysis.

Variables	Unadjusted OR(95% CI)	Adjusted OR^a^(95% CI)
MUAC <23 cm ♀/<24 cm ♂	2.6 (1.5–4.7)	2.7 (1.4–4.9)
CD4 count <350 cells/mm^3^	5.2 (2.6–10.6)	5.5 (2.6–11.6)
Previous ART interruption	5.2 (1.6–16.6)	4.2 (1.2–14.1)

Abbreviations: OR, odds ratio; MUAC, mid-upper arm circumference.

^a^ Adjusted for age and the other variables remaining in the model.

### Performance of the VLTC

A total of 299/494 (61%) had either a CD4 count <350 cells/mm^3^, a previous ART interruption, or MUAC below the gender-based threshold level; 52 of whom (17%) had VF. The NPV for determination of VF was 97% (95% CI, 94–99) with a corresponding sensitivity of 91% (95% CI, 91–97), Table 3. The specificity was moderate at 43% (95% CI, 39–48). Using the occurrence of any of the VLTC components to prompt a VL, the NNT decreased to 5.8 from 8.7 for universal testing. At higher thresholds, i.e. the occurrence of 2 or 3 VLTC components to prompt a VL, sensitivity markedly decreased (37% and 4%, respectively) and was therefore not considered in further analyses.

**Table 3.** Performance of the Viral Load Testing Criteria (VLTC) and WHO criteria.

	n/N (%)	Sensitivity (95% CI)	Specificity (95% CI)	PPV (95% CI)	NPV (95% CI)	NNT
**VLTC:**						
Any criteria	299/494 (61)	91 (81–97)	43 (39–48)	17 (16–19)	97 (94–99)	5.8
Two or more criteria	75/494 (15)	37 (24–51)	88 (84–91)	28 (20–37)	91 (90–93)	3.6
Three criteria	3/494 (1)	4 (0–12)	100 (99–100)	67 (16–96)	89 (88–91)	1.5
**WHO criteria:**						
Clinical	99/467 (21)	40 (28–54)	82 (77–85)	23 (17–31)	91 (89–92)	4.3
Immunological	79/490 (16)	45 (31–59)	88 (84–91)	32 (24–40)	92 (91–94)	3.2
Clinical and/or immunological	153/494 (31)	67 (53–79)	74 (69–78)	25 (21–30)	94 (92–96)	4.0

Abbreviations: CI, confidence interval; PPV, positive predictive value; NPV, negative predictive value; NNT, numbers needed to test.

In comparison, the combined WHO criteria indicated VF in 153/494 (31%); 38 of whom (25%) had VF. The NPV was 94% (92–96) but the corresponding sensitivity was 67% (53–79), Table 3.

The VLTC had similar sensitivity for men and women, 90 and 93% respectively, but the specificity was higher among women resulting in a NPV of 94% for men versus 99% for women, supplemental Table 2. In the multivariate age-adjusted model, including MUAC, CD4 < 350 cells/mm^3^, and previous ART interruption, the direction of associations remained the same for men and women.

### Sensitivity analysis

Two sensitivity analyses were performed. First, a complete-case analysis including only those with full datasets regarding MUAC, CD4 count, treatment interruption, and WHO stage (n = 453/494; 55/57 with VF). The sensitivity increased from 91% to 95% for the VLTC, without any change for the remaining performance indicators, supplemental Table 2. The sensitivity for the combined WHO criteria decreased slightly from 67% to 64%. Second, subjects diagnosed with active tuberculosis at inclusion (n = 88) were excluded. The sensitivity of the AC increased slightly from 91% to 94% with no change of the remaining performance indicators, supplemental Table 2. The performance of the WHO criteria did not change.

## Discussions

The discrepancy between the current recommendations for VL monitoring for all patients receiving ART and the insufficient capacity for VL testing constitutes a huge obstacle for ART programs in low-income countries, especially in view of the goal of achieving universal ART coverage among PLHIV [8,10,28]. Although resources for VL monitoring are being scaled up in high-burden countries [10,19] it is important to consider alternative evidence-based strategies to monitor patients on ART. Algorithms that assess the likelihood of VF could help target resources for viral load testing in a cost-effective manner.

We have constructed such an algorithm using data recorded prospectively and blinded, in a cohort of patients starting ART at Ethiopian health centres; a representative setting for where most PLHIV globally receive ART. We decided to use only robust parameters that have low inter- and intra-observer variability. Furthermore, we did not consider data on trends in laboratory results since such information could be lacking at peripheral clinics and criteria requiring calculations can be error-prone [29].

The Viral Load Testing Criteria (VLTC) consists of three parameters all independently associated with an increased likelihood of VF: signs of malnutrition (measured by gender-specific MUAC thresholds), CD4 count <350 cells/mm^3^, and interruption of ART since last visit. These criteria are possible to use in most decentralized care settings in low-income countries.

Mid-upper arm circumference is a well-established marker of malnutrition, which also has been associated with mortality during ART [30,31]. Furthermore, we have shown its association with virological suppression (VL<400 copies/mL) at 6 months after ART start in this cohort [23]. Given the observational nature of this study, we cannot determine the mechanisms involved in this association. It is possible that reflects impoverishment in this population, but it could also be a consequence of continued HIV replication with HIV-related wasting.

Inadequate treatment adherence has been linked with the risk of VF [14,15], but such an association was not observed in our cohort. However, VF was more common in patients with treatment interruptions. We consider this parameter to be reliable and easy to measure compared with more complex assessments of adherence level. A similar criterion was part of clinical algorithm for VF developed in Uganda [17], and associated with VF in a study from South Africa [32].

Over the last decade availability of CD4 count testing has increased as part of ART roll-out [33]. Although CD4 count measurement may not be necessary for many patients with universal access to ART [34], this technology can still be useful in settings with limited treatment coverage or limited access to VL. We used a ROC curve to determine an appropriate threshold level for CD4 count. The threshold 350 cells/mm^3^ was chosen for its high sensitivity (82%) and acceptable specificity (54%) and coincided with the median CD4 count at 12 months after ART start. Both CD4 count <100 cells/mm^3^ and CD4 count below baseline (WHO immunologic criteria) were associated with VF, but with low sensitivities at 23% and 32%, respectively. Different approaches in the use of CD4 counts to detect individuals with failing treatment has been suggested, such as risk charts [35] and CD4 gain percentile curves [36]. To keep the VLTC simple, and user-friendly we did not consider changes in CD4 count over time. However, the threshold indicating increased likelihood of VF is influenced by the CD4 count at treatment initiation. Indeed, our participants had a median CD4 count of 192 cells/mm^3^ at ART start, comparable with pre-ART counts in many African settings [37]. However, ART is now recommended for all PLHIV irrespective of CD4 counts [8], and it is likely that this will affect the performance of CD4 count data for identification of VF.

The use of algorithms will inevitably lead to some degree of misclassification. The VLTC had high sensitivity with acceptable specificity resulting in a NPV of 97%. Since the criteria should be regarded as a screening method to identify patients in need of VL testing to determine whether VF is present, sensitivity must be high. We considered construction of a scoring system based on sums of individual criteria, but since this compromised sensitivity markedly we decided to use the criteria separately.

There have been previous attempts to construct algorithms for targeted viral load testing based in Sub-Saharan Africa [15–17,35,36] and Cambodia [14]. Compared with these algorithms, the VLTC has few parameters, does not require any calculations, and only use information that can be available point-of-care. Despite this, it achieved high sensitivity in derivation. A clinical predictor score developed in Cambodia [14] achieved comparable sensitivity (78%) in a subsequent validation in Cambodia [38], but the sensitivity was low (51%) when validated in Uganda [15]. The clinical algorithms constructed in Sub-Saharan Africa (2 in Uganda and 1 in South Africa), had sensitivities ranging from 67% to76% [15–17], external validations of these algorithms are yet to be published. The sensitivity of the 2013 WHO criteria was higher in this cohort (67%) compared with previously reported data [15,16,18]. For targeting viral load testing, however, misclassification of 33% of subjects with VF cannot be accepted.

The drawback of the high sensitivity for the VLTC is its limited specificity requiring VL testing of a large proportion (61%).

The development of point-of-care testing devices has great potential in improving access to VL with reduced turnaround time [39]. However, due to limited capacity of such devices, a combination with central, high-volume testing is still needed [40]. Algorithms such as the VLTC could be considered for determination of subjects at highest risk of VF that should be for point-of-care testing, whereas remaining samples are sent to central laboratories. For such an approach, a combination of at least two criteria of the VLTC could be considered, increasing specificity to 88% for point-of-care testing.

This study was performed in health centres with nurse-based care, a setting in which most PLHIV receive their care. Data used for this study were prospectively collected from participants in a well-characterized cohort by nurse-clinicians blinded to the outcome of the study following a structured protocol. All participants were investigated for active tuberculosis at baseline. In line with a previous report from this cohort, VF was not associated with tuberculosis co-infection [23].

This study has some limitations. We defined VF as a single viral load ≥1000 copies/mL. Since some patients with a single elevated viral load level will have suppressed viremia on repeated testing [6], this definition could overestimate the rate of treatment failure. However, the VLTC is not intended to diagnose treatment failure (defined by the WHO as a VL above 1000 copies/mL at two consecutive measurements with adherence counselling in between [8]), but rather to identify patients at risk who need viral load testing. Data on drug resistance was no available for the participants of this study, information that unfortunately seldom is available in low-income settings. A proportion of subjects who started ART were excluded from analysis since a study visit with accompanying viral load was not available, which may have had some impact on the findings. In particular, a higher proportion of male participants were excluded for this reason, but few were lost to follow-up. Importantly, the VLTC has hitherto not been externally validated, which is necessary to assess it robustness before implementation in standard care. This also concerns the performance of this algorithm among ART-experienced patients, who were not included in our cohort.

**Figure 2.**
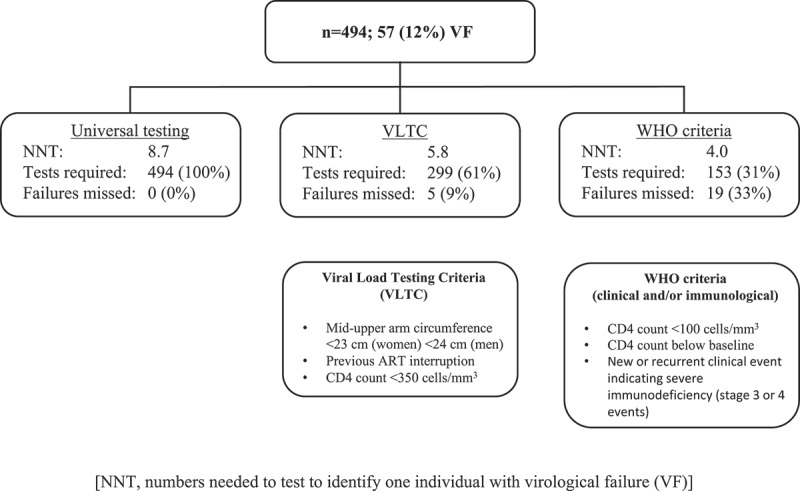
Algorithms for viral load testing.

In conclusion, the VLTC consisting of three simple-to-measure criteria, was more sensitive than the 2013 WHO criteria in determining the likelihood of VF 1 year after starting ART. The VLTC could therefore be used to rule out VF in 4/10, reducing the numbers needed to test from 8.7 (universal testing) to 5.8 for each VF identified (Figure 2). VL resources could thereby be allocated more efficiently, with few cases of missed VF.

## Supplementary Material

Supplemental_Tables.docx

## Paper context

Virological monitoring is recommended for people receiving antiretroviral therapy, but monitoring capacities are insufficient in low-income countries. The World Health Organization’s immunological and clinical failure criteria have poor performance for identification of virological failure. We present an algorithm (Viral Load Testing Criteria), that in derivation has high sensitivity and acceptable specificity to identify virological failure 12 months after treatment initiation. If externally validated, these criteria could be used to allocate viral load resources more efficiently.

## References

[1] Department of Health and Human Services Panel on Antiretroviral Guidelines for Adults and Adolescents. Guidelines for the use of antiretroviral agents in HIV-1-infected adults and adolescents. 2016 [cited 2017 8 22]. Available from: http://aidsinfo.nih.gov/contentfiles/lvguidelines/AdultandAdolescentGL.pdf

[2] European AIDS Clinical Society HIV guidelines 8.1. 2016 [cited 2017 8 22]. Available from: http://www.eacsociety.org/files/guidelines_8.1-english.pdf

[3] Haas AD, Keiser O, Balestre E, et al Monitoring and switching of first-line antiretroviral therapy in adult treatment cohorts in sub-Saharan Africa: collaborative analysis. Lancet HIV. 2015;2:e271–8. 26423252 10.1016/S2352-3018(15)00087-9 PMC4500741

[4] Phillips AN, Pillay D, Garnett G, et al Effect on transmission of HIV-1 resistance of timing of implementation of viral load monitoring to determine switches from first to second-line antiretroviral regimens in resource-limited settings. Aids. 2011;25:843–850. 21192233 10.1097/QAD.0b013e328344037a

[5] Boender TS, Kityo CM, Boerma RS, et al Accumulation of HIV-1 drug resistance after continued virological failure on first-line ART in adults and children in sub-Saharan Africa. J Antimicrob Chemother. 2016;71:2918–2927. 27342546 10.1093/jac/dkw218

[6] Bonner K, Mezochow A, Roberts T, et al Viral load monitoring as a tool to reinforce adherence: a systematic review. J Acquir Immune Defic Syndr. 2013;64:74–78. 23774877 10.1097/QAI.0b013e31829f05ac

[7] Ouattara EN, Robine M, Eholié SP, et al Laboratory monitoring of antiretroviral therapy for HIV infection: cost-effectiveness and budget impact of current and novel strategies. Clin Infect Dis. 2016;62:1454–1462. 26936666 10.1093/cid/ciw117 PMC4872286

[8] World Health Organization Consolidated guidelines on the use of antiretroviral drugs for treating and preventing HIV infection: recommendations for a public health approach. Geneva: World Health Organization; 2016. 27466667

[9] Joint United Nations Programme on HIV/AIDS. Global AIDS update 2016 Geneva; 2016 [cited 2017 8 22]. Available from: http://www.unaids.org/sites/default/files/media_asset/global-AIDS-update-2016_en.pdf

[10] Lecher S, Williams J, Fonjungo PN, et al Progress with scale-up of HIV viral load monitoring — seven Sub-Saharan African Countries, January 2015–June 2016. MMWR Morb Mortal Wkly Rep. 2016;65:1332–1335. 27906910 10.15585/mmwr.mm6547a2

[11] Roberts T, Cohn J, Bonner K, et al Scale-up of routine viral load testing in resource-poor settings: current and future implementation challenges. Clin Infect Dis. 2016;62:1043–1048. 26743094 10.1093/cid/ciw001 PMC4803106

[12] World Health Organization Guidelines for intensified tuberculosis case-finding and isoniazid preventive therapy for people living with HIV in resource constrained settings. Geneva: World Health Organization; 2011.

[13] Balcha TT, Skogmar S, Sturegard E, et al A clinical scoring algorithm for determination of the risk of tuberculosis in HIV-infected adults: a cohort study performed at Ethiopian Health Centers. Open Forum Infect Dis. 2014;1:ofu095–ofu095. 25734163 10.1093/ofid/ofu095 PMC4324227

[14] Lynen L, An S, Koole O, et al An algorithm to optimize viral load testing in HIV-positive patients with suspected first-line antiretroviral therapy failure in Cambodia. J Acquir Immune Defic Syndr. 2009;52:40–48. 19550349 10.1097/QAI.0b013e3181af6705

[15] Aboxuyannis M, Menten J, Kiragga A, et al Development and validation of systems for rational use of viral load testing in adults receiving first-line ART in sub-Saharan Africa. Aids. 2011;25:1627–1635. 21673555 10.1097/QAD.0b013e328349a414 PMC3725464

[16] Evans DH, Maskew M, Fox M, et al CD4 criteria improves the sensitivity of a clinical algorithm developed to identify viral failure in HIV-positive patients on first-line antiretroviral therapy. J Int Aids Soc. 2014;17:1–9. 10.7448/IAS.17.1.19139 PMC4165719 25227265

[17] Meya D, Spacek LA, Tibenderana H, et al Development and evaluation of a clinical algorithm to monitor patients on antiretrovirals in resource-limited settings using adherence, clinical and CD4 cell count criteria. J Int AIDS Soc. 2009;12:3. 10.1186/1758-2652-12-3 PMC2664320 19261189

[18] Rutherford GW, Anglemyer A, Easterbrook PJ, et al Predicting treatment failure in adults and children on antiretroviral therapy: a systematic review of the performance characteristics of the 2010 WHO immunologic and clinical criteria for virologic failure. Aids. 2014;28:S161–S169. 24849476 10.1097/QAD.0000000000000236

[19] Ethiopian Federal Ministry of Health Health Sector Transformation Plan - Annual Performance Report EFY 2008 (2015/2016). Addis Ababa; 2016 [cited 2017 8 22]. Available from: http://www.moh.gov.et/web/guest/-/health-sector-transformation-plan-i-annual-performance-report-arm_2016-?inheritRedirect=true

[20] National AIDS Resource Center HIV/AIDS Estimates and Projections in Ethiopia, 2011-2016. 2016 [cited 2017 8 22]. Available from: http://fitun.etharc.org/resources/finish/53-hiv-aids-estimates-and-projections/327-hiv-aids-estimates-and-projections-in-ethiopia-2011-2016

[21] Ministry of Health of Ethiopia National Guidelines for Comprehensive HIV Prevention, Care and Treatment. 2014 [cited 2017 8 22]. Available from: https://aidsfree.usaid.gov/sites/default/files/ethiopia_natl_gl_2014.pdf

[22] Balcha TT, Sturegård E, Winqvist N, et al Intensified tuberculosis case-finding in HIV-positive adults managed at Ethiopian health centers: diagnostic yield of Xpert MTB/RIF compared with smear microscopy and liquid culture. PLoS One. 2014;9:e85478. 24465572 10.1371/journal.pone.0085478 PMC3899028

[23] Reepalu A, Balcha TT, Skogmar S, et al High rates of virological suppression in a cohort of human immunodeficiency virus-positive adults receiving antiretroviral therapy in Ethiopian health centers irrespective of concomitant tuberculosis. Open Forum Infect Dis. 2014;1:ofu039–ofu039. 25734107 10.1093/ofid/ofu039 PMC4324187

[24] Mannheimer SB, Mukherjee R, Hirschhorn LR, et al The CASE adherence index: A novel method for measuring adherence to antiretroviral therapy. AIDS Care. 2006;18:853–861. 16971298 10.1080/09540120500465160 PMC2567829

[25] World Health Organization March 2014 Supplement to the 2013 consolidated guidelines on use of antiretroviaral drugs for treating and preventing HIV infection - recommendations for a public health approach. Geneva: World Health Organization; 2014.

[26] World Health Organization Haemoglobin concentrations for the diagnosis of anaemia and assessment of severity. Geneva: World Health Organization; 2011.

[27] Tang AM, Dong K, Deitchler M, et al Use of Cutoffs for Mid-Upper Arm Circumference (MUAC) as an indicator or predictor of nutritional and health- related outcomes in adolescents and adults: a systematic review Washington (DC): FHI 360/FANTA; 2013.

[28] Médecins Sans Frontières (MSF) Putting HIV and HCV to the Test: A Product Guide for Point-of-Care CD4 and Laboratory-Based and Point-of-Care Virological HIV and HCV Tests. MSF; 2015 [cited 2017 8 22]. Available from: https://www.msfaccess.org/sites/default/files/HIV_HCV_Report_Diagnostic_Guide_2015.pdf

[29] Van Griensven J, Phan V, Thai S, et al Simplified clinical prediction scores to target viral load testing in adults with suspected first line treatment failure in Phnom Penh, Cambodia. PLoS One. 2014;9:1–5. 10.1371/journal.pone.0087879 PMC3913697 24504463

[30] Reepalu A, Balcha TT, Skogmar S, et al Factors associated with early mortality in HIV-positive men and women investigated for tuberculosis at Ethiopian health centers. PLoS One. 2016;11:1–13. 10.1371/journal.pone.0156602 PMC4896420 27272622

[31] Liu E, Spiegelman D, Semu H, et al Nutritional status and mortality among HIV-infected patients receiving antiretroviral therapy in Tanzania. J Infect Dis. 2011;204:282–290. 21673040 10.1093/infdis/jir246

[32] Boulle A, Van Cutsem G, Hilderbrand K, et al Seven-year experience of a primary care antiretroviral treatment programme in Khayelitsha, South Africa. Aids. 2010;24:563–572. 20057311 10.1097/QAD.0b013e328333bfb7

[33] World Health Organization Progress report. Global health sector response to HIV, 2000–2015. Focus on innovations in Africa. Geneva: World Health Organization; 2015.

[34] Ying R, Granich RM, Gupta S, et al CD4 cell count: declining value for antiretroviral therapy eligibility. Clin Infect Dis. 2016;62:1022–1028. 26826372 10.1093/cid/civ1224 PMC5006297

[35] Koller M, Fatti G, Chi BH, et al Implementation and operational research: risk charts to guide targeted HIV-1 viral load monitoring of ART: development and validation in patients from resource-limited settings. J Acquir Immune Defic Syndr. 2015;70:e110–9. 26470034 10.1097/QAI.0000000000000748 PMC5395665

[36] Yotebieng M, Maskew M, Van Rie A. CD4+ gain percentile curves for monitoring response to antiretroviral therapy in HIV-infected adults. Aids. 2015;29:1067–1075. 26125140 10.1097/QAD.0000000000000649 PMC4487417

[37] Siedner MJ, Ng CK, Bassett IV, et al Trends in CD4 count at presentation to care and treatment initiation in Sub-Saharan Africa, 2002-2013: a meta-analysis. Clin Infect Dis. 2015;60:1120–1127. 25516189 10.1093/cid/ciu1137 PMC4366582

[38] Phan V, Thai S, Koole O, et al Validation of a clinical prediction score to target viral load testing in adults with suspected first-line treatment failure in resource-constrained settings. J Acquir Immune Defic Syndr. 2013;62:509–516. 23334504 10.1097/QAI.0b013e318285d28c

[39] Alemnji G, Onyebujoh P, Nkengasong JN Improving laboratory efficiencies to scale-up HIV viral load testing. Curr Opin HIV AIDS. 2017;12(2):165–170. 28059956 10.1097/COH.0000000000000346 PMC13081754

[40] Peter T, Ellenberger D, Kim AA, et al Early antiretroviral therapy initiation: access and equity of viral load testing for HIV treatment monitoring. Lancet Infect Dis. 2016;3099:2014–2017. 10.1016/S1473-3099(16)30212-2 PMC5745573 27773596

