# Supplementary material for: Development of an algorithm for determination of the likelihood of virological failure in HIV-positive adults receiving antiretroviral therapy in decentralized care
Source: Glob Health Action. 2017 Sep 15;10(1):1371961. doi: 10.1080/16549716.2017.1371961 (PMC5645660; doi:10.1080/16549716.2017.1371961)
Supplement: Supplemental_Tables.docx [file ZGHA_A_1371961_SM0342.docx]

Supplemental Table 1. Univariate odds ratios for virological failure (VF).

| **Variable** | **Missing data** | **non-VF**  **(n=437)** | **VF**  **(n=57)** | **OR**  **(95% CI)** | ***P*** |
| --- | --- | --- | --- | --- | --- |
| Male gender | 0 | 152 (31) | 29 (51) | 1.9  (1.1-3.4) | 0.02 |
| Age | 0 | 32 (28-40) | 36 (30-44) | 1.0  (1.0-1.1) | 0.09 |
| Karnofsky status <90% | 0 | 54 (12) | 18 (32) | 2.4  (1.3-4.4) | <0.01 |
| Body-mass index <18,5 kg/m^2^ | 9 (2) | 52 (12) | 12 (21) | 1.9  (1.0-3.9) | 0.07 |
| MUAC <23 cm ♀ / <24 cm ♂ | 10 (2) | 95 (22) | 24 (43) | 2.6  (1.5-4.7) | <0.01 |
| Baseline tuberculosis | 0 | 78 (18) | 10 (18) | 1.0  (0.5-2.0) | 0.96 |
| Adherence <95% | 2 (0) | 67 (15) | 9 (16) | 1.0  (0.5-2.2) | 0.94 |
| Previous ART interruption | 0 | 8 (2) | 5 (9) | 5.2  (1.6-16.3) | 0.01 |
| CD4 cell count <350 cells/ mm^3^ | 4 (1) | 199 (46) | 46 (82) | 5.4  (2.7-11.0) | <0.01 |
| Lymphocyte count <1100 cells/mm^3^ | 8 (2) | 61 (14) | 13 (24) | 1.9  (1.0-3.7) | 0.07 |
| Haemoglobin <11.0 g/dL | 3 (1) | 52 (12) | 11 (20) | 1.8  (0.9-3.7) | 0.11 |
| Skin rash | 0 | 17 (4) | 2 (4) | 0.9  (0.2-4.0) | 0.89 |

Abbreviations: OR, odds ratio; CI, confidence interval; MUAC, mid-upper arm circumference;

ART, antiretroviral treatment.

Data presented as n (%) or median (interquartile range) if not stated otherwise.

*P* value derived from the univariate logistic regression model.

Supplemental table 2. Performance of the Viral Load Testing Criteria (VLTC) and WHO criteria for men and women, excluding incomplete-cases, and excluding subject with baseline tuberculosis, respectively.

|  | **n/N**  **(%)** | **Sensitivity**  **(95% CI)** | **Specificity**  **(95% CI)** | **PPV**  **(95% CI)** | **NPV**  **(95% CI)** | **NNT** |
| --- | --- | --- | --- | --- | --- | --- |
| **VLTC*:** |  |  |  |  |  |  |
| Men only | 129/181  (71) | 90  (73-98) | 32  (25-40) | 20  (18-23) | 94  (85-100) | 5.0 |
| Women only | 170/313  (54) | 93  (77-99) | 49  (44-55) | 15  (13-17) | 99  (95-100) | 6.5 |
| Complete-cases | 279/453  (62) | 95  (85-99) | 43  (38-48) | 19  (17-20) | 98  (95-99) | 5.4 |
| Subjects with tuberculosis excluded | 242/406  (60) | 94  (83-99) | 45  (40-50) | 18  (16-20) | 98  (95-99) | 5.5 |
| **WHO criteria*:** |  |  |  |  |  |  |
| Men only | 68/181  (38) | 62  (42-79) | 67  (59-75) | 26  (20-34) | 90  (85-94) | 3.8 |
| Women only | 85/313  (27) | 71  (51-87) | 77  (72-82) | 24  (18-30) | 96  (94-98) | 4.3 |
| Complete-cases | 147/453  (32) | 67  (53-79) | 72  (68-77) | 25  (21-30) | 94  (92-96) | 4.0 |
| Subjects with tuberculosis excluded | 119/453  (26) | 64  (49-77) | 75  (70-80) | 25  (20-31) | 94  (92-96) | 4.0 |

Abbreviations: CI, confidence interval; PPV, positive predictive value; NPV, negative predictive valure; NNT, numbers needed to test.

* VLTC, positive if any criterion is present; WHO criteria, combined clinical and/or immunological criteria.
